# Spontaneous Coronary Artery Dissection in Patients with Autosomal Dominant Polycystic Kidney Disease: A Systematic Review of the Literature

**DOI:** 10.3390/jpm14070702

**Published:** 2024-06-29

**Authors:** Stefan Milutinovic, Abraham Bell, Predrag Jancic, Dragana Stanojevic, Abdul Hamid Borghol, Jonathan Mina, Fouad T. Chebib, Ibrahim Khambati, Ricardo O. Escarcega, Malissa J. Wood

**Affiliations:** 1 Internal Medicine Residency Program at Lee Health, Florida State University College of Medicine, Cape Coral, FL 33909, USA; smilutinovic@fsu.edu (S.M.); abraham.bell@leehealth.org (A.B.); orlando.escarcega@leehealth.org (R.O.E.); 2 Faculty of Medicine, University of Belgrade, 11000 Belgrade, Serbia; 3 Clinic for Cardiology, University Clinical Center Nis, 18000 Nis, Serbia; draganastanojevic1@gmail.com; 4 Division of Nephrology and Hypertension, Mayo Clinic, Jacksonville, FL 32224, USA; berghol.abdulhamib@mayo.edu (A.H.B.); chebib.fouad@mayo.edu (F.T.C.); 5 Department of Internal Medicine, Staten Island University Hospital, Northwell Health, Staten Island, NY 10305, USA; jonathan.mina@northwell.edu; 6 Associates in Nephrology, Cape Coral, FL 33909, USA; ikhambati@associatesnephrology.com; 7 Lee Health Heart Institute, Fort Myers, FL 33908, USA; malissa.wood@leehealth.org

**Keywords:** SCAD, ADPKD, polycystin, vascular integrity

## Abstract

Spontaneous coronary artery dissection (SCAD) is a spontaneous intimal tear of the coronary artery wall. A factor rarely associated with SCAD is autosomal dominant polycystic kidney disease (ADPKD). Using the PRISMA guidelines, we identified 10 unique cases of SCAD in ADPKD patients reported between 1998 and 2021. Ages ranged from 36 to 59 years, with an average of 44.6 years. The majority of patients were female (80%). Each case was diagnosed with a cardiovascular event: ST-elevation myocardial infarction (STEMI) in 40%, non-ST elevation myocardial infarction (NSTEMI) in 50%, and stable angina in 10%. Conservative management was used in 60% of cases. There is a significant gap in our understanding of the relationship between SCAD and ADPKD. Polycystin complex can lead to structural abnormalities in blood vessels, resulting in vascular leaks and vessel rupture. This suggests that ADPKD patients may have an elevated risk of arteriopathies, including coronary artery dissection.

## 1. Introduction

Spontaneous coronary artery dissection (SCAD) is a spontaneous intimal tear of the coronary artery wall. The recent increase in awareness and knowledge among healthcare providers has spotlighted SCAD as a significant cause of non-traumatic, nonatherosclerotic acute coronary syndrome (ACS) [1]. SCAD predominantly affects middle-aged women, accounting for over 90% of cases, and has an overall prevalence of up to 4% among patients presenting with ACS [2,3]. Notably, SCAD is a leading cause of myocardial infarction (MI) related to pregnancy (43%) and is often associated with multiple associative and contributory factors including pregnancy and the post-partum state, fibromuscular dysplasia and other vasculopathies, and physical and emotional stressors. Another factor rarely associated with SCAD is autosomal dominant polycystic kidney disease (ADPKD) [4], a prevalent hereditary renal cystic disease, which is characterized by genetic abnormalities on the polycystic-kidney-disease 1 (PKD1) locus or chromosome 4 (the polycystic-kidney-disease 2 (PKD2) locus). These loci encode for polycystin-1 and polycystin-2, respectively [5,6]. Pathogenic variants in these genes diminish polycystins’ expression and function, leading to the disease’s characteristic phenotypic features. Such features include aneurysmal changes in cranial and extracranial arteries, cardiac valvular abnormalities and left ventricular hypertrophy regardless of the presence of hypertension [7]. Heart valves are reported to be involved in a quarter of patients, most commonly presenting as mitral valve prolapse and aortic regurgitation [7].

Polycystins are vital for the structural integrity of arterial walls, as they play a crucial role in plasma membranes, especially at focal adhesions, desmosomes, and adherent junctions [8]. Although the occurrence of SCAD in patients with ADPKD has been documented, the precise nature of their association remains poorly understood due to the disease’s distinctive characteristics. This systematic review aims to synthesize existing literature to explore the potential association between these distinct diseases and contribute new insights into the extrarenal manifestations of ADPKD.

## 2. Materials and Methods

We performed a systematic literature review of all SCAD and ADPKD cases utilizing Preferred Reporting Items for Systematic Reviews and Meta-Analyses (PRISMA) guidelines, searching the Midline (via PubMed search engine), Embase, and Scopus databases. This study is registered with ResearchRegistry, and the unique identifying number is reviewregistry1828. All reported articles were analyzed from database inception until 1 April 2023. In total, 25 original articles were identified using a combination of MeSH and non-MeSH terms: (“polycystic kidney, autosomal dominant”[MeSH Terms] OR “autosomal dominant polycystic kidney*”[Text Word]) AND (“dissection, blood vessel”[MeSH Terms] OR “Coronary vessel anomalies”[MeSH Terms] OR “spontaneous coronary artery disease”[Text Word] OR “spontaneous coronary artery dissection*”[Text Word] OR “coronary artery dissection”[Text Word]). We identified 1 more case from another resource, but that case was excluded since it was published in Portuguese. We included cases with proven ADPKD and SCAD. The diagnosis of ADPKD was established based on clinical or genetic testing. The diagnosis of SCAD was established via coronary angiography. We excluded cases where the diagnosis of ADPKD or SCAD was not certain or there was a plausible alternative diagnosis. Furthermore, duplicate articles, articles in a language other than English, abstracts without comprehensive case descriptions, and narrative reviews were all excluded.

**Figure 1:**
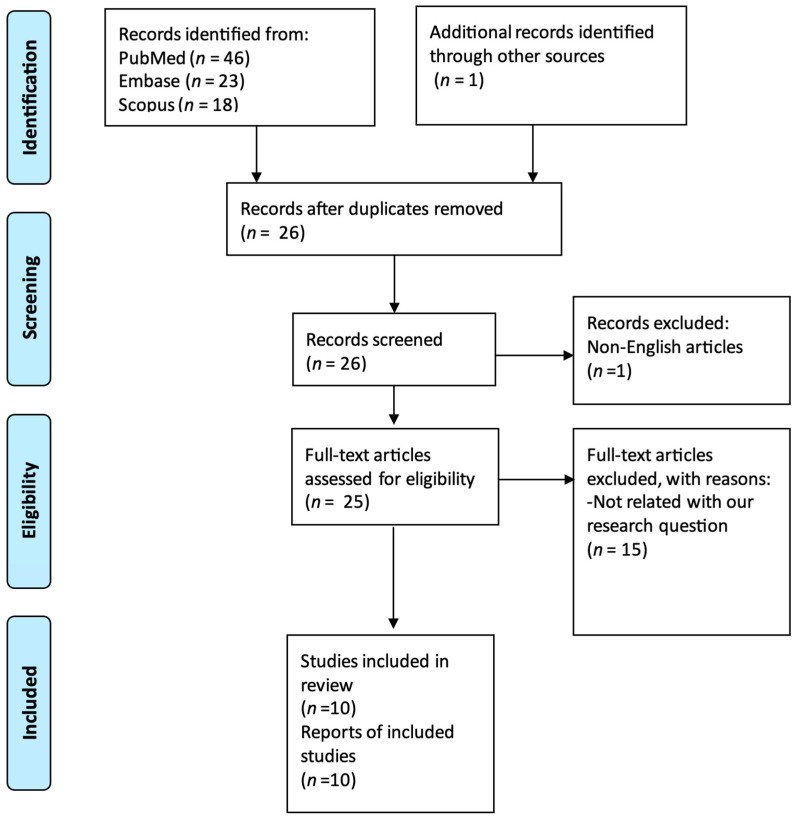
PRISMA flow chart.

Two authors (S.M. and A.B.) independently and blindly identified and selected titles, abstracts, and full texts in the database search. Discrepancies in the selected articles were resolved by the senior author (P.J.). Additionally, the reference list of selected articles was searched to identify any additional cases for inclusion in accordance with previously established selection criteria. The flow chart of detailed article selection and the final cases included in the analysis are illustrated in Figure 1.

An Excel table was constructed, and for each case, we extracted patients’ demographic data, co-morbid conditions, presenting symptoms, physical exam findings, laboratory and imaging findings (including ECG, echocardiography, and angiography findings, amongst other imaging findings), treatment options, complications, and outcomes.

Data were analyzed using descriptive statistics and expressed as mean ± standard deviation for continuous data, or as frequencies and percentages for categorical data. Percentages for categorical data were determined based on the total count of cases that provided specific categorical information. Non-reported values were excluded from calculations.

## 3. Results

### 3.1. Demographics and Comorbidities

Our systematic review identified 10 unique cases of SCAD reported between 1998 and 2021 (Table 1) [9,10,11,12,13,14,15,16,17,18]. 

The patients, all adults, ranged in age from 36 to 59 years, with an average age of 44.6 years (standard deviation 6.4). The majority were female (80%). The most common comorbidity was a history of liver cysts, reported in 40% of cases, followed by hypertension (HTN) in 30%. Other noted conditions included being overweight (20%), and individual cases of contraceptive use, migraines, chronic kidney disease (CKD), subdural hematoma, and liver transplantation. Eight cases explicitly reported taking both medical and family history; however, none of the patients reported genetic connective tissue disorder such as fibromuscular dysplasia (FMD), Ehlers–Danlos syndrome, Marfan syndrome, or Loeys–Dietz syndrome. Ninety percent of the patients were confirmed to have ADPKD prior to their ACS presentation, whereas one patient received their diagnosis after experiencing SCAD. There were no reports of SCAD occurrences in the patients’ families. No cases reported the ADPKD genotype or Mayo classification system.

### 3.2. Presentation and Evaluation

**Figure 2:**
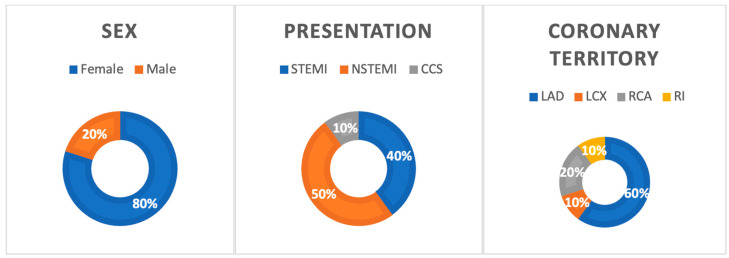
Sex, presentation, and coronary territory of SCAD; STEMI—ST segment elevation myocardial infarction; NSTEMI—non-ST segment elevation myocardial infarction; CCS—chronic coronary syndrome; LAD—left anterior descending artery; LCX—left circumflex artery; RCA—right coronary artery; RI—ramus intermedius.

All patients presented with chest pain or chest discomfort. Additionally, two patients exhibited neurologic symptoms, including left-sided facial droop and dizziness. An identifiable stressor was reported in 60% of the cases, evenly divided between exercise (30%) and emotional stress (30%). Diagnostic evaluations for all patients included EKG and troponin level measurements. Briefly, 8 patients (80%) underwent echocardiography, while invasive coronary angiography (ICA) was performed on all 10 patients. Each case was diagnosed with a cardiovascular event: ST-elevation myocardial infarction (STEMI) in 40%, non-ST elevation myocardial infarction (NSTEMI) in 50%, and stable angina in 10%. Only 20% of patients had coronary artery disease (CAD) on angiography. The dissections most commonly affected the left anterior descending artery (60%), followed by the right coronary artery (20%), with the left circumflex and ramus intermedius each involved in one case (Figure 2).

### 3.3. Treatment, Complications, and Outcomes

Treatment approaches for the patients varied, with the most common strategy being conservative management using medication in 60% of the cases (six patients). For the other four patients, an invasive method involving percutaneous coronary intervention (PCI) and the placement of drug-eluting stents was preferred. Notably, one patient in this invasive treatment group initially received thrombolytic therapy. There were reports of conditions worsening after the initial treatment in two instances. Follow-up assessments were conducted at 8.1 ± 8.4 months, on average, after treatment, ranging from 1 to 24 months. Remarkably, there were no fatalities reported during the follow-up period; all patients were stable and asymptomatic.

## 4. Discussion

Spontaneous coronary artery dissection is characterized by an injury to the intimal vessel wall, leading to the development of an intramural hematoma that can result in significant ischemia and myocardial infarction [3,4]. Despite SCAD’s lack of association with traditional cardiovascular risk factors like hypertension and atherosclerosis, several unique risk factors have been proposed, including extreme physical effort, emotional stressors prior to the event, and fluctuations in sex hormones [3,19,20,21] This last factor is particularly noteworthy given the prevalence of pregnancy-associated SCAD, the most common cause of myocardial infarction in pregnant patients, and the predominance of female patients in SCAD cases [22,23,24,25]. Although a familial pattern of SCAD has been observed, suggesting genetic inheritance, the search for specific genes has been inconclusive [26]. However, patients with vascular-predominant genetic disorders such as Ehlers–Danlos syndrome, Marfan syndrome, and fibromuscular dysplasia have been reported to experience SCAD [27,28,29]. This association supports the theory that vascular wall fragility contributes to SCAD, yet our systematic review did not find any patients with a history of these genetic disorders. Between 5 and 8% of SCAD patients are reported to carry deleterious gene variants associated with heritable connective tissue disorders [30,31].

Estimating the true prevalence of SCAD is somewhat difficult, given the relative unfamiliarity of this disease and the fact that over 70% of cases lack classic angiography findings [32]. A study from Germany showed that among 3803 patients with coronary artery disease, 1.1% were found to have a diagnosis of SCAD [33]. In addition, the incidence was higher in patients presenting with acute coronary syndrome and ranged between 2.9% and 4.2% [33]. A newer study by Lobo et al. found that SCAD was diagnosed in 1% of STEMI patients with 19% of these being females ≤ 50 years old [34]. Given the increased awareness and utilization of intra-vascular imaging, SCAD incidence has increased 10-fold in the last two decades [35]. A retrospective analysis utilizing the National Inpatient Sample Database between 2010 and 2017 found that the overall crude incidence of SCAD increased from 4.95/1,000,000 to 14.81/1,000,000 [36]. The same study found that the incidence was highest among white people, in the highest household income quantile, and in the West region of the US.

The occurrence of SCAD in patients with ADPKD, linked to pathogenic variants in the *PKD1* and *PKD2* genes, underscores the complexity of SCAD’s etiology. Despite its rarity, the relationship between SCAD and ADPKD remains poorly understood and has been scarcely reported in the literature. A population-based cohort study in the United States involving 66,360 SCAD patients found a significantly higher prevalence of ADPKD in SCAD patients compared with non-SCAD acute coronary syndrome patients (*p* < 0.001) [37]. The authors utilized the National Inpatient Sample (NIS) database between 2004 and 2015. Among the study population, there were 60 (0.09%) patients with ADPKD. Lastly, there is a possibility that the NIS overestimates SCAD diagnosis by including many non-spontaneous coronary artery dissections that are wrongly coded, making the true percentage of patients with both ADPKD and SCAD likely higher. The presence of molecularly identifiable disorders was further examined in a prospective Massachusetts General Hospital SCAD registry database [31]. This study included 44 patients who underwent genetic testing, between July 2013 and September 2017. Most of these patients were females (85%) with a mean age of 43.6 ± 9.6. The presence of traditional risk factors for atherosclerotic coronary artery disease was low: 27.9% of patients had a history of smoking, 37.2% had hypertension, 23.5% had hyperlipidemia, and only 4.6% had diabetes mellitus. The average age at the first SCAD event was 45.3 ± 9.4. The genetic testing showed that six patients (8.2%) had an identifiable underlying genetic disease that could explain dissection. Of those, one patient had an ADPKD. The patient enrolled with ADPKD and SCAD in this study experienced their first SCAD event at age 38, after having a vertebral dissection at age 34. Additionally, two more patients had mutations in the PKD1 and PKD2 genes, which were considered variants of unknown significance. Interestingly, 25% of the patients enrolled in the study had fibromuscular dysplasia. However, none of the six patients with an underlying genetic disorder had this disease. 

The association between ADPKD and coronary artery aneurysms, which may pose an increased risk for SCAD, has been documented since the early 1990s through case reports and series, indicating a higher prevalence of coronary aneurysms in this patient group compared with the general population with suspected coronary heart disease [38,39,40]. Elfanish et al. reported a giant 4.7 cm left main coronary artery in an ADPKD patient [38]. Moreover, a case series of 32 patients found aneurysmal coronary vessels in 11 angiograms (34%) [41]. A similar retrospective analysis from Sweden found that the prevalence of coronary aneurysm was 13%, still higher than that in the general population with suspected coronary heart disease (1.5%) [42]. A retrospective analysis of end-stage renal disease (ESRD) patients on hemodialysis with ADPKD found that their coronary arteries had larger diameters compared with those of non-ADPKD ESRD patients [43]. The average diameters of the left main coronary artery, LAD, and RCA were significantly higher in ADPKD patients; however, the LCX did not differ between the two groups. The pathophysiological mechanisms associated with ADPKD may have contributed to these findings. Although no dissections were reported during the follow-up period, it is postulated that the larger diameter of coronary arteries and aneurysmatic changes could lead to coronary events. This hypothesis, however, requires further investigation.

**Figure 3:**
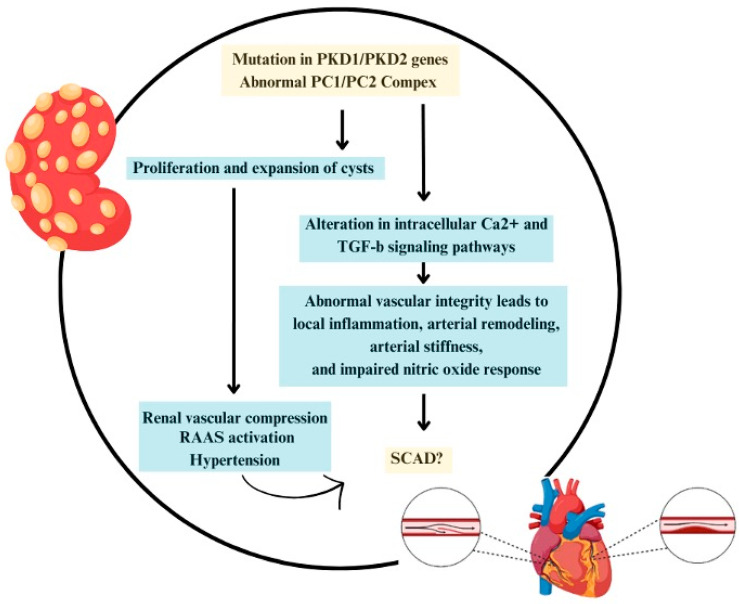
Pathophysiology behind spontaneous coronary artery dissection and ADPKD.

Our systematic review uncovered nine case reports and one case review study, comprising 10 patients diagnosed with ADPKD and presenting with SCAD, highlighting the need for further investigation into the links between these conditions. In the context of ADPKD, a notable interplay occurs between vascular wall protein composition and hypertension (Figure 3). 

Pathogenic variants in *PKD1* and *PKD2* proteins not only lead to renal cystic formation with subsequent renal parenchymal destruction but also cause dysregulation in vascular wall signaling pathways, doubly influencing HTN development [44]. While hypertension is not traditionally considered a risk factor for SCAD, it is a prevalent condition among ADPKD patients, often occurring earlier than in the general population [45]. Notably, hypertension in these patients is preceded by endothelial dysfunction (ED). Endothelial dysfunction is mediated by a reduction in vasodilatory substances, including nitric oxide with increased production of pro-vasoconstrictive, pro-inflammatory, and pro-thrombotic mediators that can lead to vascular wall derangements and early atherosclerosis. Turkmen et al. examined coronary flow velocity reserve in 30 normotensive ADPKD patients with normal kidney function by using transthoracic Doppler echocardiography before and after dipyridamole infusion [46]. These patients were compared with 30 healthy individuals, and matched for blood pressure, weight, blood glucose, and lipid profile. Patients with ADPKD were found to show significant impairments of the coronary flow velocity reserve, which was found to be associated with cardiovascular events during long-term follow-up. Interestingly, this dysregulation, coupled with dysregulation in the vascular wall signaling pathway, may affect vascular wall stability, potentially contributing to SCAD development.

Polycystic-kidney-disease 1 and *PKD2* play essential roles in maintaining vascular wall integrity, suggesting that pathogenic variants in these genes could increase the risk of vascular-related events [47]. Polycystin-1 (PC1), a product of the PKD1 gene, is an 11-transmembrane protein, has structural characteristics reminiscent of a G-coupled protein receptor, and is found typically on the plasma membrane and primary cilia [48]. In contrast, polycystin-2 (PC2) is typified by six-transmembrane spans (these six-transmembrane spans show some homology to the last six-transmembrane spans of PC1) and can assemble as a tetramer where it can form a calcium-dependent cation-selective channel [49,50,51,52]. Both polycystins are involved in complex cellular roles within the vascular wall, including the activation of smooth muscle cells leading to vasoconstriction and the activation of endothelial cells resulting in vasodilation via nitric oxide (NO) production [53,54,55]. This dual mechanism is believed to be mediated by primary cilia on the endothelium, composed partly of polycystin 1 and 2 proteins, which respond to shear stress by activating pathways that result in NO production [56,57]. 

Further investigation into PKD1/2 complex defects leads to alterations in intracellular Ca^2+^, cyclic AMP, and TGF-b signaling pathways, underscoring the proteins’ critical and complex roles in vascular wall homeostasis [58,59,60]. Notably, ADPKD patients without HTN have also been found to have elevated inflammatory markers (IL6, TNF alpha, hs-CRP) [61]. Animal studies, including a murine model, have suggested a link between polycystin dysfunction and hypertension [62]. Similarly, human studies have observed decreased NO production in ADPKD patients, indicating a possibly dysregulated response to vascular wall shear stress [63]. Additionally, a proposed association between ADPKD and polycystic ovary syndrome (PCOS) via insulin resistance and PAI-1 activity suggests a shared pathway influencing connective tissue remodeling [64]. Knockout studies targeting *PKD1* and *PKD2* have demonstrated global vascular damage and blood vessel rupture in mice, further emphasizing the potential impact of these proteins on vascular integrity [65].

**Figure 4:**
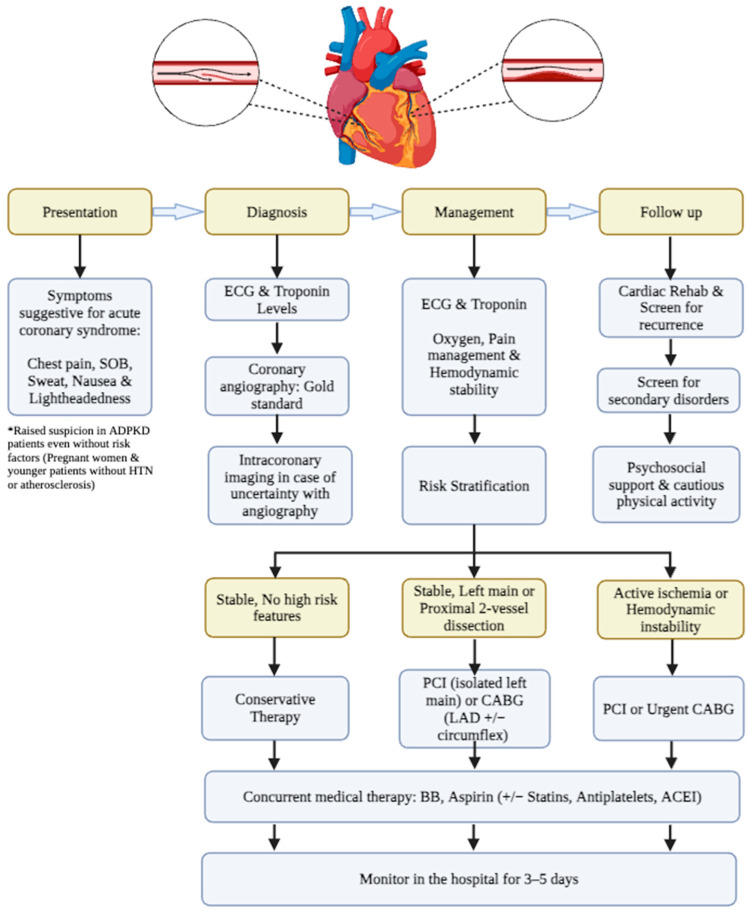
Management algorithm of SCADs in ADPKD; PCI—percutaneous coronary intervention; CABG—coronary artery bypass graft; LAD—left anterior descending artery. *Raised suspicion in ADPKD patients even without risk factors (Pregnant women & younger patients without hypertension or atherosclerosis.

Our findings are consistent with previous SCAD reports in non-ADPKD patients, demonstrating several key similarities. Regarding demographic characteristics, the highest percentage of patients with SCAD and ADPKD includes females with a mean age of less than 50. This aligns with other studies on SCAD, with almost 90% of these patients being perimenopausal women [1,4,66]. Hormonal changes, in addition to decreased vascular integrity in ADPKD patients, could explain this finding. Additionally, LAD has been the most commonly affected coronary artery, which is also the case in non-ADPKD patients [67]. The presence of atherosclerotic risk factors in SCAD patients was believed to be less prevalent than that in those with ischemic cardiomyopathy. However, recent data indicate that these factors are present in older SCAD patients, showing no significant differences compared with other ischemic cardiomyopathies [1,4,66]. In our review, ADPKD-SCAD patients were found to have similar atherosclerotic risk factors as a non-ADPKD group. Hypertension was only reported in 30% of patients, and coronary artery disease was found in just 20% of coronary angiographs. The absence of conventional risk factors for myocardial infarction might lead to a delay in diagnosing SCAD. Therefore, maintaining a high level of suspicion, particularly in ADPKD patients, is crucial. Utilizing high-sensitivity troponins alongside coronary computed tomography angiography (CCTA) can be beneficial for patients experiencing chest pain and classified as being at low-to-intermediate risk of coronary events, as previously suggested (Figure 4) [10]. 

However, invasive coronary angiography (ICA) remains the most important diagnostic tool for SCAD. When the structure of the arterial wall is unclear, intravascular imaging modalities such as IVUS or optical coherence tomography (OCT) should be considered. The typical signs of SCAD on coronary angiography include multiple radiolucent cavities and extraluminal contrast agent retention, suggesting that there may be a spiral dissection or intraluminal filling defects. SCAD coronary angiography is classified as follows: type 1 refers to the typical signs of multiple radiolucent cavities or tube wall filling with the contrast agent; type 2 refers to multiple stenosis of different lengths and degrees; type 2A refers to the cause of normal diffuse arterial stenosis defined by the proximal and distal segments; type 2B refers to diffuse stenosis extending to the distal end of the artery; and type 3 refers to focal or tubular stenosis, usually less than 20 mm in length, similar to atherosclerosis [68]. Short-term complications and outcomes were favorable in all of the patients, without a single recorded death, which is in agreement with prior data [67].

As these are case reports, there is a notable inconsistency in data reporting in terms of accurately characterizing the stage of ADPKD, blood pressure control, family history of SCAD, etc. However, it is intriguing to note that a prior review has indicated a higher incidence of familial burden related to the aneurysmal involvement of blood vessels, including the coronary arteries, which could increase the risk of SCAD [11]. Further investigation is necessary to better understand this relationship and the potential role of family history in SCAD development. 

Additionally, future research on ADPKD and SCAD should focus on elucidating the underlying mechanisms that link these two diseases. Large-scale, multicenter studies conducted to gather comprehensive data on the prevalence and clinical characteristics of SCAD in ADPKD patients are needed. Genetic studies may also uncover specific mutations or genetic predispositions that contribute to the occurrence of SCAD in ADPKD patients. Furthermore, advanced imaging techniques and biomarker studies could help in early detection and risk stratification. Ultimately, a deeper understanding of this association will lead to better diagnostic, preventive, and therapeutic approaches, improving patient outcomes.

## 5. Conclusions

There is a significant gap in our understanding of the relationship between spontaneous coronary artery dissection and autosomal dominant polycystic kidney disease. It is believed that a defect in the polycystin complex can lead to structural abnormalities in blood vessels, resulting in vascular leaks and vessel rupture. This suggests that ADPKD patients may have an elevated risk of arteriopathies, including coronary artery dissection. Healthcare providers should maintain a high level of suspicion of SCAD in ADPKD patients presenting with possible symptoms of cardiac ischemia thorough medical history and diagnostic testing, including the performance of an electrocardiogram, high-sensitivity troponin testing, and non-invasive imaging like CCTA, which could aid in diagnosis. 

## 6. Limitations

This study has limitations. Our systematic review included only 10 cases, representing a small sample size. The review was restricted to articles published in English and sourced from three databases, potentially overlooking high-quality cases that did not meet our selection criteria. While the results of this review are hypothesis-generating, the observational nature of the data means that larger, more comprehensive studies are needed to clarify the connection between SCAD and ADPKD. It would be advantageous to investigate whether or not individuals categorized with higher Mayo Classification Classes exhibit an elevated risk for SCAD, or conversely, whether those who achieve targeted blood pressure control demonstrate a lower risk of SCAD.

## Figures and Tables

**Table 1:** Characteristics and clinical course of reported SCAD cases in ADPKD patients.

Reference/Year	Sex/Age	Important Past Medical History	Clinical Presentation	Stressor/Culprit	ADPKD/GFR/CKD on Presentation	ACS/Coronary Artery	Management
Klingenberg-Salachova et al., 2012 [9]	41, M	Subdural Hematoma	Chest pain	/	Normal GFR	STEMI/LAD	Medical
Itty et al., 2009 [10]	43, F	HTN, Overweight, ADPKD	Central chest pain	Exercise	CKD 2	NSTEMI/LAD	PCI
Grover et al., 2016 [11]	52, F	ADPKD	Chest pressure radiating to left arm. Transient left facial droop for 5 min.	/	/	NSTEMI/RCA	Medical
Afari et al., 2013 [16]	46, F	HTN, ADPKD	Substernal chest pain, dyspnea, and diaphoresis, radiating to shoulder and neck	Psychosocial	/	NSTEMI/RI	Medical
Bento et al., 2016 [18]	41, F	ADPKD, Overweight	1-week progressive precordial discomfort with radiation to both upper limbs	/	Normal GFR	STEMI/RCA	PCI
Lee et al., 2011 [12]	59, M	ADPKD	Mild chest discomfort	Hiking	Normal GFR	CCS/LAD	PCI
Qian et al., 2021 [13]	46, F	HTN, ADPKD	Chest pain lasting 1 day radiating to the back of left shoulder	Emotional Stress	CKD 4	NSTEMI/LCX	Medical
Bobrie et al., 1998 [14]	36, F	ADPKD, OCP use	Chest pain whilst jogging	Exercise	/	STEMI/LAD	Medical
Verlaeckt et al., 2019 [15]	44, F	History of liver transplantation, ADPKD	Episodes of chest pain radiating to the left arm	Steroids, Emotional Stress	CKD 3A	NSTEMI/LAD	Medical
Basile et al., 2009 [17]	38, F	ADPKD	Chest pain	/	Normal GFR	STEMI/LAD	PCI

M—male; F—female; HTN—hypertension; ADPKD—autosomal dominant polycystic kidney disease; OCP—oral contraceptive pills; GFR—glomerular filtration rate; CKD—chronic kidney disease; STEMI—ST segment elevation myocardial infarction; NSTEMI—non-ST segment elevation myocardial infarction; CCS—chronic coronary syndrome; LAD—left anterior descending artery; LCX—left circumflex artery; RCA—right coronary artery; RI—ramus intermedius.
